# Ipsilateral brain cavernoma under scleroderma plaque: a case report

**DOI:** 10.11604/pamj.2019.32.13.15288

**Published:** 2019-01-08

**Authors:** Vimal Raj Nitish Gunness, David Munoz, Pablo González-López, Nabeel Alshafai, Agdaliya Mikhalkova, Julian Spears

**Affiliations:** 1 Neurosurgery Department St Michael’s Hospital, Toronto, Ontario, Canada; 2 Department of Laboratory Medicine, St Michael’s Hospital, Toronto, Ontario, Canada; 3 Neurosurgery Service, Alicante University General Hospital, Alicante Institute for Health and Biomedical Research (ISABIAL-FISABIO Foundation), Alicante, Spain; 4 Neurosurgery Department, Royal Commission Hospital, Al Lulu Road, Fanateer, Al Jubail, Saudi Arabia; 5 Neurosurgery Department, Academic Teaching Hospital of Feldkirch, Feldkirch, Austria

**Keywords:** Scleroderma, cavernoma, adult, ipsilateral, brain, skin incision

## Abstract

Scleroderma is a rare disease of unknown etiology, which is characterized by thickening and hardening of skin due to an increased collagen production. A 44-year-old female patient with a scleroderma on the scalp known by our department, also presented an ipsilateral brain lesion since 2015, which was showing growth without any clinical symptomatology and the patient wanted the lesion to be removed. This atypical lesion underneath the scleroderma shows that diagnosis can be missed without brain imaging and biopsy.

## Introduction

Localized scleroderma encompasses the conditions of linear scleroderma (LS) (extremity and facial), plaque or circumscribed morphea, pansclerotic and generalized morphea. By definition, localized scleroderma involves the skin and the underlying tissue [1]. The most commonly described brain lesions in LS *'en coup de sabre'* are intracranial calcifications, which are characteristically ipsilateral to the skin lesions and appear as hypo intense white matter lesions on T2-weighted magnetic resonance imaging (MRI) [2-4]. Cerebral angiograms and magnetic resonance angiogram studies may typically show vascular involvement suggestive of vasculitis. Reports of cerebral aneurysms and other vascular malformations, as brain cavernomas [1, 5, 6], exist and could represent late sequelae of a vasculitic process. To our knowledge there have been no case reports published showing a brain carvernous malformation ipsilateral to the scleroderma and beneath it in the adult population. However, we found 2 case reports in pediatric series.

## Patient and observation

**Figure 1:**
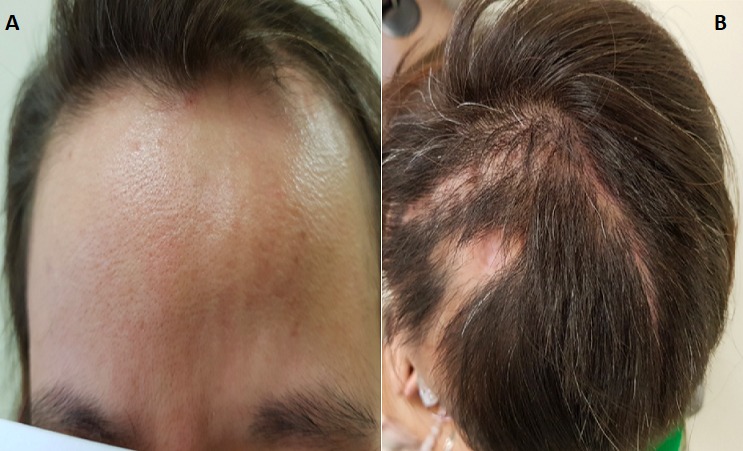
(A) Linear scleroderma; (B) incision line and localized scleroderma plaque

**Figure 2:**
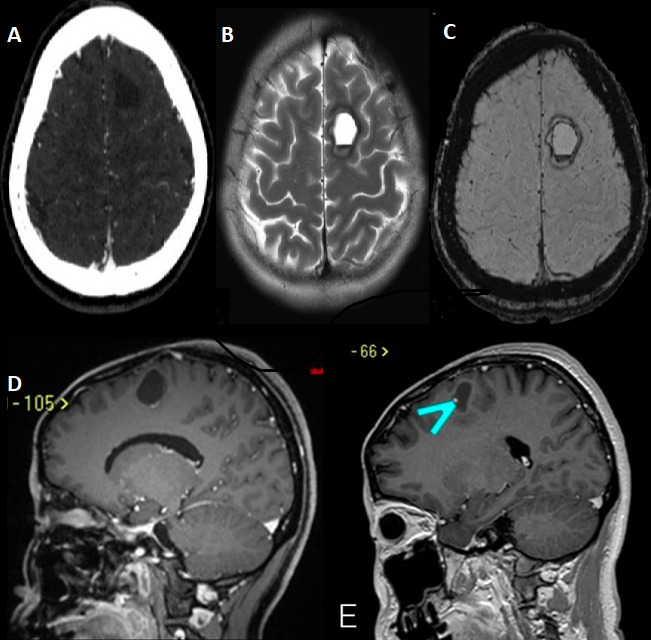
(A) axial CT angiogram shows no vascular malformation; (B) T2 axial image showing a fluid level with small amount of hematocrit level; (C) axial cuts showing SWI sequence shows few punctate foci of blooming artifacts; (D) sagittal section with contrast showing enhancement along inferior aspect of lesion; (E) sagittal cut showing a post contrast enhancement of a nodule (*blue arrow*)

**Figure 3:**
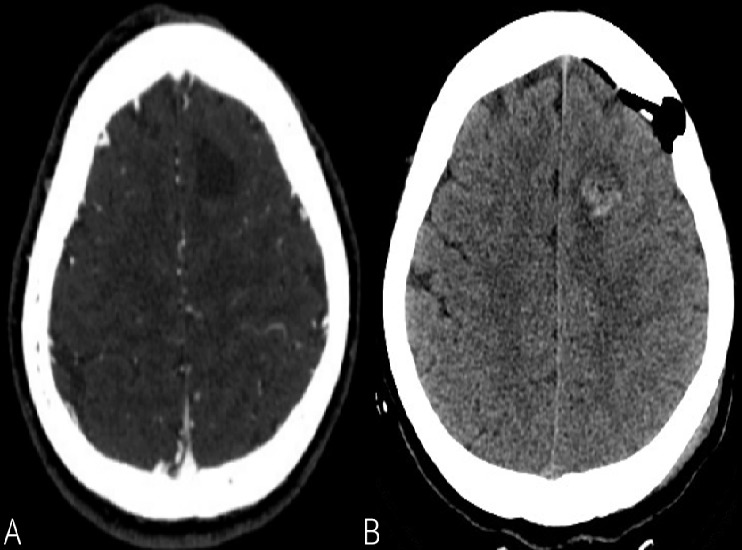
(A) angio CT axial cuts does not show any vascular malformation; (B) post-surgical CT showing cyst was removed completely

**Figure 4:**
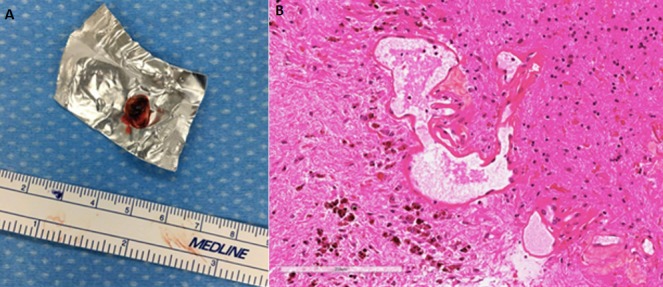
(A); 7mm nodule which was contrast enhancing after surgical removal, (B); histopathologic analysis after surgical removal of the nodule. Thin walled vessels of irregular outline amidst chronic reactive gliosis and hemosiderin deposition in the neuropil

A 44-year-old of Asian origin female was followed up in our department since December 2015. She was referred by her family doctor due to a left frontal lobe cystic lesion. The patient has been following herbal treatment for her scleroderma since she was 3 years old in China. The patient had an MRI of her brain done in China, because a Chinese medicine practitioner in Toronto told her that she might have something in the brain due to scleroderma. The scleroderma was localised in the left frontal side of her scalp (Figure 1). She did not complain of any symptoms at that time. After observing the MRI, she was taking methotrexate because of her scleroderma in 2015 in Toronto. Her past medical history showed a very limited scleroderma involving her left scalp and a little bit of the left side of her face. There was no skin involvement elsewhere and she denied any symptoms or history of systemic sclerosis. She just highlighted she suffered malnutrition when she was a child. She referred allergy to penicillin. She was followed with CT angiogram and an MRI was done in February 2016. The CT angiogram did not show any vessel abnormality. Figure 2 shows the features that were discovered on the MRI sequences. The patient had a second MRI done on May 2016, which showed a stable lesion. She was visited in the clinic and she did not complain of any new symptoms. She had a repeat MRI every year and on May 2017 the intraaxial lesion showed a mild increase in size of the lesion. The post contrast MRI showed a tiny enhancing nodule along the lateral margin of the lesion (Figure 2). When the patient was seen again in the clinic on September 2017, she complained of mild sensory changes on her right side that have been around for several years and so, wanted the intraaxial lesion to be removed; however, this symptom had not changed recently as she referred. Neurological exam at that time showed GCS 15, motor strength was 5/5 on both upper and lower extremities and reflexes were present, no focal neurological changes were discovered. No sensory deficiency was noticed. The head of plastic surgery was asked to review the case as well as to attend the operating room for an evaluation of the superficial approach for optimum scalp incisional planning and we decided we would stay to the medial side with the opening of the scalp to include a broad-based craniotomy flap that contained this abnormality of the scalp to optimize healing. The patient was informed about the risks of surgery, accepting them and signing the informed consent. The patient was subsequently brought in the operation theatre for a left frontal craniotomy. The incision was planned with the help of plastic surgeons to incorporate the abnormality in the scalp flap with a broad base (Figure 1). Intraoperatively, we used neuronavigation and ultrasound to locate the tumor which was placed below the scalp lesion and skull defect. Brain needle was then used under direct guidance to tap the cyst with hemorrhagic fluid which looked chronic and sent for cell count and analysis. The cyst wall was resected which looked like there was a deposit of hemosiderin. Two areas of nodularity were seen, one was consistent with the ultrasound and was removed. It looked like a thrombosed vein or hemangioblastoma nodule principally. All specimens were sent to pathology for evaluation. Then, the cavity was inspected under the microscope view and no other nodule was visible. A gross total resection was achieved (Figure 3). The examination (Figure 4) of the histopathology sections showed white matter containing multiple blood vessels of irregular outline with paper-thin walls lacking smooth muscle and lined by a single layer of endothelial cells. Some of the vessels were obsolete and replaced by hyalinised connective tissue. The white matter demonstrated severe piloid gliosis with numerous Rosenthal fibers. Multiple clusters of hemosiderin-laden macrophages were scattered throughout. CD34 highlighted the thin walled blood vessels. No neoplastic abnormalities were present. Final diagnosis was a cavernous angioma.

## Discussion

The patient had a mild increase in the lesion without any neurological deficiency and she asked for surgical treatment. Surgical indication was probably controversial at that time, although there was an increase in tumour size. Considering that the lesion was growing and she is a young patient, we carried forward with the surgery. This present case is remarkable for several reasons, but the main one is because of the uncommon mixed presentation. To our knowledge, this is the third case report presenting with a cavernoma ipsilateral to the localized scleroderma. Fain *et al*. reported 2 cases in children [1]. The localized scleroderma without systemic involvement itself, is a rare entity [7]. Cavernous angiomas are clusters of abnormally dilated blood vessels. The cavernoma was located beneath the sclerodermic lesion. There is no case reported in the literature where we can find this unique feature, moreover showing a tinning of the scalp in the left frontal region. There is a theory about the pathogenesis arguing that vasculature is the primary target [8]. The pathogenic mechanisms behind neurological dysfunction associated with Linear Scleroderma and Parry-Romberg Syndrome (progressive hemifacial atrophy) remain unclear, although several theories have been proposed. Perhaps the most plausible and widely accepted explanation is the “neurovasculitis hypothesis” based on focal MRI findings and angiographic evidence of CNS vascular changes [9-11]. There is the belief that a previous infection, particularly due to *Borrelia Burgdorferi*, has been implicated in Europe and Japan, but not confirmed in the United States [12, 13]. Genetic participation in pathogenesis appears to be relatively weak, since only a 4.7% concordance between twins has been observed [14] and family studies revealed only 1.6% frequency among first-degree relatives [4, 15]. However, several groups have identified polymorphisms in potential candidate genes involved in immune regulation, such as BANK1, C8orfl3-BLK, IL-23R, IRF5, STAT4, TBX21 and TNFSF4, which may underlie the pathogenesis of systemic sclerosis [16, 17]. Intriguingly, many of these polymorphisms are shared with other rheumatic diseases, such as systemic lupus erythematosus. Another unique feature is that this is a reported case in a female adult with brain cavernoma, while the previous two reported cases have been described in paediatric population [1]. There are a couple of manuscripts, published by Moko *et al.* [18] and K. Terstegge [19], showing that asymptomatic patients with LS who received CNS imaging showed that 82-100% (n = 13) had exclusively ipsilateral lesions in the brain. None of the lesions were brain cavernous angiomas. This is the first case ever, where the brain cavernoma was resected in a patient in whom we had to find the way to make the incision avoiding the linear scleroderma.

## Conclusion

This case report highlights that patients diagnosed of a Linear Scleroderma should have an MRI of the brain to exclude any underlying pathology and to be monitored yearly to check the lesion´s behaviour.

## Competing interests

The authors declare no competing interests.
